# The Immunogenicity of HLA Class II Mismatches: The Predicted Presentation of Nonself Allo-HLA-Derived Peptide by the HLA-DR Phenotype of the Recipient Is Associated with the Formation of DSA

**DOI:** 10.1155/2017/2748614

**Published:** 2017-02-26

**Authors:** Vadim Jucaud

**Affiliations:** Terasaki Foundation Laboratory, 11570 W Olympic Blvd., Los Angeles, CA 90064, USA

## Abstract

The identification of permissible HLA class II mismatches can prevent DSA in mismatched transplantation. The HLA-DR phenotype of recipients contributes to DSA formation by presenting allo-HLA-derived peptides to T-helper cells, which induces the differentiation of B cells into plasma cells. Comparing the binding affinity of self and nonself allo-HLA-derived peptides for recipients' HLA class II antigens may distinguish immunogenic HLA mismatches from nonimmunogenic ones. The binding affinities of allo-HLA-derived peptides to recipients' HLA-DR and HLA-DQ antigens were predicted using the NetMHCIIpan 3.1 server. HLA class II mismatches were classified based on whether they induced DSA and whether self or nonself peptide was predicted to bind with highest affinity to recipients' HLA-DR and HLA-DQ. Other mismatch characteristics (eplet, hydrophobic, electrostatic, and amino acid mismatch scores and PIRCHE-II) were evaluated. A significant association occurred between DSA formation and the predicted HLA-DR presentation of nonself peptides (*P* = 0.0169; accuracy = 80%; sensitivity = 88%; specificity = 63%). In contrast, mismatch characteristics did not differ significantly between mismatches that induced DSA and the ones that did not, except for PIRCHE-II (*P* = 0.0094). This methodology predicts DSA formation based on HLA mismatches and recipients' HLA-DR phenotype and may identify permissible HLA mismatches to help optimize HLA matching and guide donor selection.

## 1. Introduction

Human Leukocyte Antigen (HLA) incompatible transplantation frequently leads to the formation of HLA donor-specific antibodies (DSAs). DSAs are now considered to be the main cause of allograft loss [1, 2], since they are associated with an increased risk for antibody-mediated rejection (AMR), poor long-term survival, and chronic rejection [1, 3–5]. To that end, the identification of permissible HLA mismatches—not inducing antibody formation—has been essential for mismatched transplantation, as it is clear that not all HLA mismatches are immunogenic and lead to the formation of DSA [6, 7].

The development of DSA requires the activation of the transplant recipient's adaptive immune system against allogeneic HLA (allo-HLA), a process known as the indirect allorecognition pathway (reviewed in [8, 9]). This alloreactive response involves both B and T cell compartments of the immune system, particularly the cognate CD4+ T cell helper function, to produce long-lasting IgG alloantibodies [8, 10, 11]. Indeed, the indirect allorecognition initiates when the B cell receptors (BCR) of allospecific B cells bind to their cognate allo-HLA. This binding induces B cells to internalize and process the allo-HLA into allo-HLA-derived peptides (allo-HLApep). Only certain allo-HLApep are then loaded onto the recipient's HLA class II molecules and presented on the B cell surface. The presented allo-HLApep can be recognized by the T cell receptors (TCR) of the recipient's allo-HLA-specific follicular helper CD4+ T cells (allo-T_FH_); these cells have undergone thymic selection, which creates a pool of allo-T_FH_ that expresses TCR with a low affinity for “self” peptides/HLA class II complex and a high affinity for “nonself” ones [12, 13]. Only the costimulatory signal provided by allo-T_FH_ induces B cell proliferation and differentiation into memory B cells and IgG-secreting plasma cells, both specific for the allo-HLA [9, 14, 15].

The immunogenic potential, or immunogenicity, of allo-HLA molecules remains to be fully elucidated. Nonetheless, allo-HLA molecules have to carry two important immunogenic domains to induce a humoral immune response [16]. The first domain is a B cell epitope that is specific for a unique BCR expressed on the surface of allospecific B cells. The second domain, a T cell epitope recognized by allo-T_FH_, is the allo-HLApep presented by the recipient's HLA class II antigens on the surface of allospecific B cells. Several computational approaches have been developed to characterize and clarify the immunogenicity of allo-HLA (reviewed in [17]).

B cell epitopes (or HLA antibody epitopes) can be identified using the Duquesnoy HLAmatchmaker method, which is a quantitative tool used to distinguish polymorphic eplets of donor/recipient HLA mismatches in order to identify actual and potential antigenic determinant specificities of human allo-HLA and murine monoclonal antibodies [18–20]. HLAmatchmaker evaluates the eplet mismatch load of a transplant, and both transplant survival and DSA formation were correlated with high eplet mismatch load [20–22]. This is supported by Dankers et al., who reported that DSA formation correlated with the number of amino acid triplet differences [23]. Moreover, characterizing the frequencies of epitope-specific allo-HLA antibodies provides a measure of the relative immunogenicity of HLA eplet mismatches [24]. Similarly, the group of Komoliaptsis V. have shown that the number and physiochemical properties (hydrophobicity and electrostatic charge difference) of amino acid mismatches were correlated with the presence and level of DSA [25–28]. While the relative immunogenicity of HLA antigens was assessed using the antigenicity of HLA epitopes, it does not fully reveal their immunogenicity [7].

On the other hand, the HLA class II phenotype, HLA-DR in particular [29, 30], of the recipient contributes to the immunogenicity of HLA mismatches by presenting allo-HLA-derived T cell epitopes and thus influencing CD4+ T cell and B cell interactions [31]. Potential T cell epitopes can be identified using an Internet-based prediction tool (NetMHCIIpan 3.1, developed by Nielsen M. and his team) to determine the binding affinity of nonamer cores from any protein sequence [32], with the highest performance comparable to other available methods [33]. The group of Spierings E. developed an allogeneic HLA-derived, predicted indirectly recognizable HLA epitopes, HLA class II-presented (PIRCHE-II) method to identify the number of nonself peptides with high predicted binding affinities (IC_50_ < 1000 nM) for a specific HLA class II antigen [34]. The allogeneic HLA-derived PIRCHE-II number was correlated with the development of HLA antibodies in kidney and pancreas transplantation [34, 35], as well as during pregnancy [36], suggesting that allo-HLA-derived T cell epitopes are critical for evaluating the immunogenicity of HLA.

The immunogenicity of HLA mismatches, assessed using a B cell epitope or a T cell epitope approach, had limitations. Indeed, in many mismatches with low eplet mismatch load, physiochemical property differences, or PIRCHE-II numbers, DSA can be formed and vice versa. The overlap in the distribution of the aforementioned HLA mismatch characteristics between DSA producer and DSA nonproducers suggests that other factors are involved in the immunogenicity of HLA mismatches [37].

To my knowledge, the self component of HLA mismatches has not been investigated before, yet it may be a factor influencing the immunogenicity of allo-HLA. Because HLA molecules have both unique and shared sequences of amino acids, allospecific B cells, which processed an allo-HLA, can present self and/or nonself allo-HLApep depending on their binding affinity for the recipient's HLA class II expressed. The thymic selection of T cells creates a repertoire of allo-T_FH_ cells expressing specific TCRs with a low affinity for self peptides/HLA class II complex and a high affinity for nonself ones [12, 13]. Unlike self allo-HLApep, the recognition of nonself allo-HLApep provides adequate costimulation for the proliferation and differentiation of B cells into allo-HLA-specific IgG-secreting plasma cells. Therefore, I hypothesize that comparing the highest predicted binding affinity of nonself and self allo-HLApep for a transplant recipient's HLA class II antigens may distinguish immunogenic HLA mismatches (which induce the formation of DSA) from nonimmunogenic ones.

## 2. Materials and Methods

### 2.1. Patients with HLA Class II Mismatches

Six renal transplant recipients, with defined HLA class II genotype and IgG DSAs previously characterized at the Terasaki Foundation Laboratory by Dr. El-Awar, were used in this study [38]. Briefly, all patients were transplanted between 2002 and 2010 and had undergone AMR. The HLA-DRB1, HLA-DQA1, and HLA-DQB1 molecular typing were performed using Luminex polymerase chain reaction sequence specific oligo probe hybridization (PCR-SSO) technology (One Lambda, Canoga Park, CA, USA) for all recipient/donor pairs. All patients had IgG DSA detected (MFI > 1000) at the time of AMR using the Luminex platform with single HLA antigen beads (One Lambda, Canoga Park, CA, USA). All the HLA-DQ DSAs (DQA1 and DQB1) had their epitope defined previously [38], and all of the epitopes were located on either the alpha chain or the beta chain, but not to the HLA-DQ heterodimer. The total number of HLA class II mismatches was 25 (including DRB1, DQA1, and DQB1 molecules only).

### 2.2. Methods to Evaluate Donor HLA-DRB1, HLA-DQB1, and HLA-DQA1 Mismatch Scores

#### 2.2.1. HLAmatchmaker

This program (“HLA-DRDQDP matching for up to 1000 cases (v02)” downloaded from http://www.epitopes.net/downloads.html) was used to evaluate the total eplet mismatch score (EpMS)—antibody verified and others—of each of the HLA class II mismatches, for each individual chain (DRB1, DQB1, and DQA1) separately [39, 40].

#### 2.2.2. PIRCHE-II

This analysis was attempted by manually using the Internet-based NetMHCIIpan 3.1 server predication tool (found on http://www.cbs.dtu.dk/services/NetMHCIIpan) as described by the group of Otten et al. [34] to evaluate the number of PIRCHE-II for each mismatch. Briefly, the amino acid sequence of each HLA class II mismatch (full-length mature protein comprised of the following: extracellular domain, transmembrane domain, and cytoplasmic tail) was used to predict the binding affinity of all 15-mer nonself peptides to the recipients' HLA-DR antigens' peptide groove. This prediction was made after inter- and intralocus amino acid sequence alignment comparison, and all the nonself 15-mer peptides with high predicted binding affinity (IC_50_ < 1000 nM) were identified. Only the unique nonself nonamer core sequences that aligned to the binding groove of HLA-DR antigens were counted as a PIRCHE-II.

#### 2.2.3. HLA Class II Immunogenicity Algorithm

This program is freely available to download from http://www.hlaimmunogenicity.org/download/Cambridge_HLA_Class_II_Immunogenicity_Algorithm.xls and was used to determine the amino acid mismatch score (AMS), the hydrophobicity mismatch score (HMS), and the electrostatic mismatch score (EMS) for each HLA class II mismatch [25].

### 2.3. Prediction of the Binding Affinity of Allo-HLA-Derived Peptide to Recipients' HLA Class II Antigens

**Figure 1:**
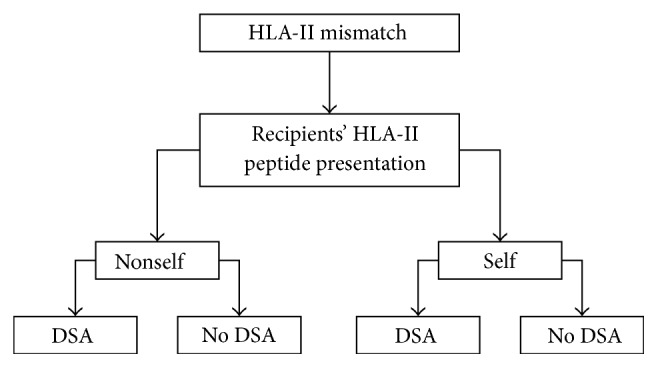
Classification of each HLA mismatch based on the presence of DSA and the nature (self or nonself) of the predicted peptide with highest affinity for the recipient's HLA class II phenotype.

With the “NetMHCIIpan 3.1” Internet-based predication tool, the amino acid sequence of each HLA mismatch (extracellular domains only, in the FASTA format found on http://www.hla.alleles.org [41]) was used to predict the binding affinity of all allo-HLApep sequences to the recipients' HLA class II antigens' peptide groove. The recipients' HLA class II antigens analyzed were HLA-DR and all possible permutations of DQA1 and DQB1 chains for HLA-DQ. For example, for a hypothetical recipient with DQA1^*∗*^X1/^*∗*^X2∖DQB1^*∗*^X3/^*∗*^X4, all the following HLA-DQ heterodimers were analyzed for their allo-HLApep binding affinity: DQA1^*∗*^X1∖DQB1^*∗*^X3; DQA1^*∗*^X1∖DQB1^*∗*^X4; DQA1^*∗*^X2∖DQB1^*∗*^X3; DQA1^*∗*^X2∖DQB1^*∗*^X4. Three allo-HLApep peptide lengths were examined: 9 mers, 12 mers, and 15 mers. The prediction output values of the binding affinity of allo-HLA-derived peptide sequences are given as follows: (i) nM IC_50_ values, where IC_50_ < 50 nM are considered high affinity, <500 nM intermediate affinity, and <5000 nM low affinity, and (ii) percentile rank (% rank) of a peptide, which is generated by comparing the peptide's score against the scores of 200,000 random natural peptides (% rank < 2%: strong binder; % rank < 10%: weak binder). After making inter- and intralocus amino acid sequence alignment comparisons, all the allo-HLApep were classified as self when they were identical to the recipients' and as nonself when they were different. As shown in Figure 1, each mismatch was classified based on whether it led to the formation of DSA or not and whether a self or nonself allo-HLApep was predicted to have the highest binding affinity for the recipient's HLA class II antigens. Self presentation was defined as follows: only self allo-HLApep with highest binding affinities for all of the recipient's HLA class II antigens; meanwhile nonself presentation was defined as follows: at least one of the recipient's HLA class II antigen having the highest binding affinity for nonself allo-HLApep.

### 2.4. Statistical Analysis

All statistical analyses were done using STATA 13. All data were tested for normality using the Shapiro-Wilk *W* test, and the Shapiro-Francia *W* test. Analysis for significance was performed using the *t*-test, with equal variance for mean comparison. Fisher's exact test was used to analyze 2 by 2 contingency tables, and the accuracy, specificity, and sensitivity were reported for the same contingency tables. Two-tailed *P* values less than 0.05 were considered significant.

## 3. Results

### 3.1. Patients and HLA Class II Mismatches

The six transplant cases analyzed had 3 to 5 HLA class II mismatches (HLA-DRB1, HLA-DQA1, and HLA-DQB1 molecules) and 2 to 4 DSAs (Table 1). The total number of mismatches was 25 (9 HLA-DRB1, 6 HLA-DQA1, and 10 HLA-DQB1), where 17 led to the formation of DSA (3 anti-HLA-DRB1, 6 anti-HLA-DQA1, and 8 anti-HLA-DQB1) and 8 did not (6 anti-HLA-DRB1, 0 anti-HLA-DQA1, and 2 anti-HLA-DQB1). Only one patient (Tx ID 21) produced DSA against all of his mismatched HLA class II antigens, whereas all other patients did not produce DSA against all their respective mismatches.

### 3.2. HLA Class II Mismatch Scores

The EpMS (antibody verified and others) and number of PIRCHE-II, HMS, EMS, and AMS are listed in Table 2 for all the mismatches. The EpMS ranged from 3 to 16 (antibody verified: 0–9; others: 1–11), the number of PIRCHE-II from 1 to 18, the HMS from 0.9 to 33.999, the EMS from 0.529 to 31.23, and the AMS from 3 to 21. For all loci combined (Table 3(a)) or separate (Tables 3(b) and 3(c)), there are no significant differences in the EpMS (antibody verified and others), HMS, EMS, and AMS between mismatches that led to the formation of a DSA and those that did not. In contrast, the numbers of PIRCHE-II were significantly higher in mismatches that led to the formation of DSA compared to those that did not for all loci combined (*P* = 0.0094, Table 3(a)), but not for the separate loci (Tables 3(b) and 3(c)).

### 3.3. Predicted Allo-HLA-Derived Peptides Presented by HLA-DRB1 Antigens of the Recipients

Table 4 shows the predicted binding affinity of allo-HLApep to their respective recipient's HLA-DR antigens. Each self or nonself allo-HLApep is characterized by its amino acid (AA) position within the whole allo-HLA molecule and its IC_50_ value and percentile rank (% rank). The same analysis was done for recipient's HLA-DQ antigens, where all possible DQA1 and DQB1 chain permutations were analyzed (data not shown).

Eight HLA class II mismatches did not lead to the formation of DSA (nonbold and nonunderlined mismatches in Table 4), of which five (mismatches ID 1, 11, 12, 19, and 24) had their respective recipient's HLA-DRB1 antigens binding with the highest and higher affinity (lowest IC_50_ and percentile rank values, bolded values in Table 4) for 9-mer self allo-HLApep compared to nonself ones. Of the remaining 3 (mismatches ID 4, 17, and 22), at least one of their respective recipient's HLA-DRB1 antigens had the highest and higher affinity for nonself allo-HLA-derived peptides compared to self ones.

In the HLA class II mismatches that led to the formation of DSA (bold and underlined mismatches in Table 4), fifteen (mismatches ID 2, 3, 5–9, 13–16, 18, 20, 23, and 25) had at least one of their respective recipient's HLA-DRB1 antigens binding with the highest and higher affinity for nonself allo-HLA-derived peptides compared to self ones. Of the remaining 2 (mismatches ID 10, 21), their respective recipient's HLA-DRB1 antigens had the highest and higher affinity self allo-HLA-derived peptides (9 mers) compared to nonself ones. The highest binding affinity (IC_50_) and percentile rank of the predicted self or nonself peptides for the recipients' HLA-DR antigens are below 5000 nM and 5%, respectively, and thus they could all be potential T cell epitopes (Table 4).

### 3.4. Prediction of DSA Formation: Influence of the Peptide Length and HLA Class II Phenotype

Table 5 shows the classification of each mismatch according to the criteria described in the Materials and Methods and the analysis presented in Table 4. Three peptide lengths (9 mers, 12 mers, and 15 mers) and the predicted HLA-DR and/or HLA-DQ presentation were analyzed for their association with DSA formation. Only the HLA-DR presentation of 9-mer peptides is significantly associated with DSA formation (*P* = 0.0169), in contrast to HLA-DQ or the combined HLA-DR/DQ presentation of 9-mer, 12-mer, and 15-mer peptides (Table 5). If the recipient's HLA-DR antigen has the highest affinity for nonself peptide derived from the mismatch then a DSA will likely be generated against it. The formation of DSA, given the presentation of nonself allo-HLA-derived peptide, can be predicted with an accuracy of 80%, a sensitivity of 88%, and a specificity of 63%.

## 4. Discussion

This study reveals that comparing the predicted binding affinity of HLA class II mismatched-derived self or nonself peptides helps distinguishing immunogenic HLA class II mismatches that lead to the formation of DSA from nonimmunogenic ones after HLA mismatched kidney transplantation. Indeed, there is a significant association between the predicted presentation of nonself peptide and the presence of DSA in the serum of kidney recipients. This immunologic evaluation of donor-recipient pairs may be useful in optimizing HLA matching and in guiding donor selection by determining potentially nonimmunogenic HLA mismatches, that is, permissible HLA mismatches.

Although the self component of HLA mismatches provides new insight for the understanding of the complex underlying mechanism of HLA antibody development, the computational approach described in this study has some limitations. Indeed, the evaluation of the immunogenicity of allo-HLA cannot rely on the characterization of T cell epitopes alone, as the recognition of B cell epitopes initiates the humoral response to allo-HLA [17]. For example, mismatch ID 17 did not lead to the formation of DSA, while nonself allo-HLApep had the highest binding affinity for the recipient's HLA-DR antigens. However, mismatch ID 17 carries a total of 6 eplet mismatches, but none are antibody verified; therefore, this mismatch may not be antigenic and may not result in the formation of DSA.

Another limitation relies on the complexity of HLA-DQ heterodimers, where both DQA1 and DQB1 chains are polymorphic and can be mismatched; thus both can contribute to the antigenicity of HLA-DQ antigens. For example, mismatch ID 10 led to the formation of anti-DQA1^*∗*^06:01 DSA but the DQA1-derived-peptides that presented with highest affinities were only self. Their associated allo-DQB1 chains (DQB1^*∗*^03:01/03:03) had nonself peptides binding with highest affinity for the recipient's HLA-DR antigens and with higher affinity than the self DQA1-derived-peptides (Table 4). In a similar fashion, mismatch ID 19 (DQB1^*∗*^06:04), which did not lead to the formation of DSA, had self DQB1-derived peptides binding with highest affinity for the recipient's HLA-DR antigens and with higher affinity than the nonself DQA1-derived-peptides. For mismatch ID 24 (DQB1^*∗*^06:03), which did not lead to the formation of DSA and self peptides were binding with highest affinity for the recipient's HLA-DR antigens, the associated mismatched DQA1 had nonself peptides binding with highest affinity. In this case, only the expression of the HLA-DQ heterodimer comprised of both mismatched DQA1 and DQB1 (DQA1^*∗*^02:01∖DQB1^*∗*^06:03) may lead to the formation of DSA against DQB1^*∗*^06:03, but not if the mismatched DQB1 chain is expressed in association with the matched DQA1 chain. Therefore, to predict the immunogenicity of HLA-DQ mismatches, both alpha and beta chains have to be taken into account, as both can carry peptide sequences recognized as nonself by the recipient (the same is true for antibody epitopes [42, 43]).

The predictive power of this analysis can be improved if the sample size is increased and if the complete HLA class II typing is available. Indeed, the HLA typing of each donor/recipient pair for HLA-DRB3, HLA-DRB4, HLA-DRB5, and DP alleles was not available for this study. This limitation prevented a complete interlocus comparison between donor and recipient, especially for DRB1 antigens. The only mismatches that did not produce DSA but involved nonself peptides that were predicted to be presented were HLA-DRB1 mismatches (ID 17 and 22). Perhaps the nonself peptides predicted to be presented could potentially be self peptides that are shared by the recipient's HLA-DRB3, HLA-DRB4, or HLA-DRB5 antigens.

Furthermore, it can be noted that mismatches ID 4 and 16 were identical. Indeed, TxID 18 and TxID 33 had the same HLA class II typing (DRB1^*∗*^11:01/^*∗*^16:01, DQB1^*∗*^03:01/^*∗*^05:02, and DQA1^*∗*^01:02/^*∗*^05:05), an HLA-DRB1^*∗*^07:01 mismatch, and at least one of their HLA-DRB1 antigens had the highest affinity for a nonself allo-HLApep. However, only TxID 33 produced an anti-HLA-DRB1^*∗*^07:01 DSA, but not TxID 18. Therefore, mismatch ID 4 may have the potential to lead to the formation of a DSA, provided the allograft remained longer in the recipient. More direct evidence is needed, however.

This study evaluated the HLA class II (HLA-DR and HLA-DQ) presentation of B cells. Although B cells can be expected to present allo-HLA-derived peptides with all HLA class II antigens (DRB, DQ, and DP) [37, 44], the predicted presentation of recipients' HLA-DR antigens only, and not HLA-DQ or both HLA-DR and HLA-DQ, is associated with the formation of DSA. Therefore, this study suggests that the HLA-DR phenotype is associated with the production of IgG DSA, as previously described [29, 30, 34]. Interestingly, the increased cell surface expression of HLA-DQ and/or HLA-DP (the antigen-presenting phenotype of B cells) was observed in autoimmune diseases (rheumatoid arthritis and psoriatic arthritis) and is possibly associated with the presentation of self antigens [44]. Coincidently, HLA-DQ antigens were often predicted to present self allo-HLA peptides (as shown in Table 5). Evaluating the antigen presentation ability of DRB3, DRB4, or DRB5 and DP antigens remains to be elucidated in the context of transplantation.

One interesting finding is that the prediction of DSA formation is significantly associated with the presentation of 9-mer and not with 12-mer or 15-mer peptides. HLA class II antigens have a binding groove that is open at both ends, which allows the binding of peptides that extend beyond the groove and can accommodate peptides of varying lengths (typically 12–18 mers) and possibly whole proteins [45]. However, the core binding motif of HLA class II antigens, which anchors the peptide in the groove, is approximately nine amino acids long [46]. In addition, TCR interaction with the HLA class II/peptide complex requires different contact sites that span the HLA class II alpha and beta chains and across the peptide [47]. Furthermore, changes in the nonamer core sequences that aligned to the binding groove of HLA class II molecules may result in critical effects on peptide contacts and interactions with the TCR [48]. Therefore, when 9-mer allo-HLApep are analyzed, every core binding motif is evaluated separately in contrast to 12- or 15-mer peptides, where different peptides can have the same core binding motif. Similarly, PIRCHE-II relies upon the distinction between identical and nonidentical peptides based upon the exact nonamer sequence that was predicted to occupy the binding groove [34]. Yet the underlying reasons that 9-mer peptides predict the formation of DSA remains to be elucidated.

Surprisingly, the EpMS, HMS, EMS, and AMS were not statistically different between mismatches that led to the formation of DSA and the ones that did not, in contrast to previous reports. Although a trend was seen, where mismatches that led to the formation of DSA had higher EpMS, HMS, EMS, and AMS than mismatches that did not produce DSA, the sample size may have influenced the statistical significance. Nevertheless, HLAmatchmaker, a structurally based matching program, evaluates the possible target of IgG DSAs, and, similarly, Komoliaptsis et al. assessed the number and physiochemical properties of amino acid mismatches. Both approaches focused on the structural basis of antibody-antigen interactions and thus elucidated the antigenicity of HLA molecules. In contrast to the present study, both groups found a correlation between the number and physiochemical characteristics of antigenic determinant mismatches and the incidence of epitope-specific HLA DSA [20–22, 24–28]. Although the immunogenicity of HLA molecules is dependent on the mismatched amino acid residues between the donor and the recipient and on the number and physiochemical properties of these amino acid mismatches that are accessible on the molecular surface of HLA, only the antigenicity of HLA molecules are reported, not the immunogenicity or the humoral response to a specific allo-HLA. In contrast, there was a significant difference between the numbers of PIRCHE-II in mismatches that led to the formation of DSA compared to those that did not for all loci combined; this lends support to the idea that the immunogenicity of HLA class II mismatches is dependent on the recipient's HLA-DR presentation of self or nonself allo-HLA-derived peptides and may be an important factor in the IgM to IgG DSA isotype switch. Lastly, a conclusion drawn by Otten et al. [34] about the antigenicity (B cell epitopes) and immunogenicity (T cell epitopes) of HLA mismatches, being independent parameters in supporting the formation of DSA, is reinforced by this study.

**Figure 2:**
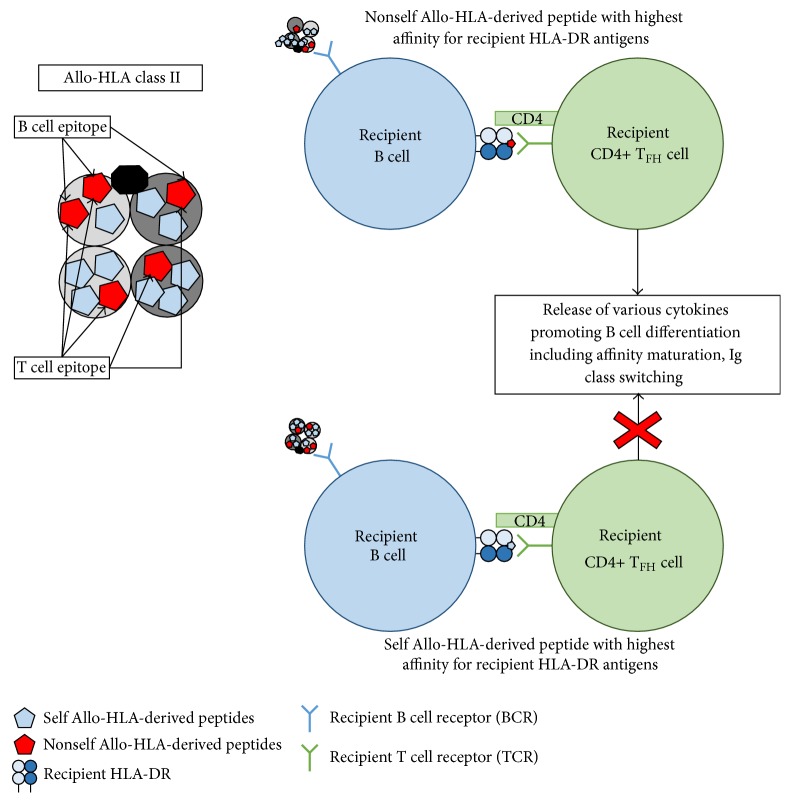
The indirect allorecognition pathway of allo-HLA class II by B cells and the presentation of self or nonself allo-HLA-derived peptide influence CD4+ T_FH_ cells helper function to lead to the formation of DSA. HLA mismatches can become immunogenic when they carry both a B cell epitope and a T cell epitope. B cell epitopes are located on the molecular surface of HLA, whereas T cell epitopes can be located anywhere, exposed or cryptic. B cells expressing a BCR specific for an allo-HLA can capture, internalize, and process the whole allo-HLA class II molecule and then present allo-HLA-derived peptides on the cell surface. Only when CD4+ T_FH_ cells recognize nonself antigenic determinants presented by B cells does the release of various cytokines promote B cell differentiation. This, in turn, may induce affinity maturation and Ig class switching, which eventually leads to the formation of DSA.

The indirect allorecognition pathway and the thymic selection of CD4+ T cells are the central components underlying the analysis of this study.  Figure 2 describes the process by which B cells capture and present allo-HLA molecules to CD4+ T cells. When nonself allo-HLA-derived peptides are presented to CD4+ T cells, the release of various cytokines promotes B cell differentiation into anti-HLA IgG-secreting plasma cells [8]. This process requires allo-HLA molecules to be antigenic (antibody reactive) and immunogenic (ability to elicit an immune response); that is, there has to be a domain recognized by B cells and a domain recognized by T cells, respectively [16]. The B cell domain, referred to as an HLA antibody epitope, should be located on the molecular surface of allo-HLA only, whereas the T cell domain, referred to as a T cell epitope, can be found anywhere, exposed or cryptic. Indeed, for HLA-I antigens, the *α*3 domain and the N-terminal part of the *α*1 domain seem to be enriched in T cell epitopes (described as PIRCHE-II), where HLA antibody epitopes (described as eplets) are rare [34]. Moreover, eplets recognized with high frequency by allo-antibodies are generally expressed on the top of the HLA molecule, in contrast to eplets recognized with low frequency that are in less accessible positions [24].

The dynamics of the HLA class II presenting pathways and the processing of allo-HLA molecules through different compartments of the endosomal/lysosomal pathway are a very complex mechanism and remain to be fully elucidated [49]. The cleavage, or proteolysis, of allo-HLA molecules into small antigenic peptides is dependent on the catalysis of different proteases [50–52], although HLA molecules are known to be resistant to protease degradation [53]. The cleavage pattern of HLA molecules by different proteases should help to identify whether certain predicted T cell epitopes (allo-HLApep) can be produced or not. An integrated approach to epitope analysis has been developed [54] and should be used in the context of transplantation by combining it with the use of databases (such as SYFPEITHI) listing peptide sequences which are known to be presented HLA class II antigens [55]. T cell epitopes can be destroyed if they contain the cleavage site of any protease [56, 57], and it could explain why certain mismatches (ID 4, 17, and 22) that were predicted to present nonself peptides did not produce a DSA, and one mismatch (ID 21) that was predicted to present self peptides led to the formation of a DSA. Lastly, it is possible that relying solely on the peptides with highest affinity for the recipient's HLA class II antigens may carry a risk of missing relevant immunogenic peptides when both self and nonself peptides have similar binding affinities for the same HLA class II antigen. It has been documented that peptide immunogenicity correlates with both its dissociation rate from and its affinity for HLA class I molecules [58, 59], and the same may be true for HLA class II molecules. However, if their binding affinity difference is large enough then this risk may be minimized.

In summary, the approach described in this paper relies on the indirect allorecognition pathway to predict the formation of DSA. The predicted presentation of nonself allo-HLA-derived peptides shows a relationship with the presence of DSA in the serum of kidney recipients. The evaluation of the immunogenicity (self or nonself peptide presentation) and antigenicity (antibody epitopes) of HLA mismatches requires the molecular typing of all HLA-I and HLA class II loci, which is not a standard practice in most organ transplantations. But this approach can benefit mismatched transplantation by optimizing donor-recipient matching and selecting permissible HLA mismatches with low immunogenic potential in order to minimize the appearance of DSA.

## Figures and Tables

**Table 1:** Transplant cases: HLA-DR and HLA-DQ mismatches and DSA.

Tx ID	Recipient HLA typing	HLA allele mismatches	Mismatch ID	DSA
13 3 mismatches 2 DSAs	DRB1^*∗*^04:05	DRB1^*∗*^11:04	1	No
	DRB1^*∗*^14:01			
	DQB1^*∗*^03:02	DQB1^*∗*^03:01	2	Yes
	DQB1^*∗*^05:03			
	DQA1^*∗*^01:01	DQA1^*∗*^05:05	3	Yes
	DQA1^*∗*^03:01			

18 3 mismatches 2 DSAs	DRB1^*∗*^11:01	DRB1^*∗*^07:01	4	No
	DRB1^*∗*^16:01			
	DQB1^*∗*^03:01	DQB1^*∗*^02:02	5	Yes
	DQB1^*∗*^05:02			
	DQA1^*∗*^01:02	DQA1^*∗*^02:01	6	Yes
	DQA1^*∗*^05:05			

21 4 mismatches 4 DSAs	DRB1^*∗*^07:01	DRB1^*∗*^08:01	7	Yes
	DQB1^*∗*^02:01	DQB1^*∗*^03:01	8	Yes
	DQB1^*∗*^02:02	DQB1^*∗*^03:03	9	Yes
	DQA1^*∗*^02:01	DQA1^*∗*^06:01	10	Yes

22 5 mismatches 3 DSAs	DRB1^*∗*^11:01	DRB1^*∗*^07:01	11	No
	DRB1^*∗*^14:07	DRB1^*∗*^15:01	12	No
	DQB1^*∗*^05:02	DQB1^*∗*^02:02	13	Yes
	DQB1^*∗*^05:03	DQB1^*∗*^06:02	14	Yes
	DQA1^*∗*^01:01	DQA1^*∗*^02:01	15	Yes
	DQA1^*∗*^01:02			

33 5 mismatches 3 DSAs	DRB1^*∗*^11:01	DRB1^*∗*^07:01	16	Yes
	DRB1^*∗*^16:01	DRB1^*∗*^13:01	17	No
	DQB1^*∗*^03:01	DQB1^*∗*^02:02	18	Yes
	DQB1^*∗*^05:02	DQB1^*∗*^06:04	19	No
	DQA1^*∗*^01:02	DQA1^*∗*^02:01	20	Yes
	DQA1^*∗*^05:05			

35 5 mismatches 3 DSAs	DRB1^*∗*^04:03	DRB1^*∗*^07:01	21	Yes
	DRB1^*∗*^13:02	DRB1^*∗*^15:01	22	No
	DQB1^*∗*^03:02	DQB1^*∗*^02:02	23	Yes
	DQB1^*∗*^06:04	DQB1^*∗*^06:03	24	No
	DQA1^*∗*^01:02	DQA1^*∗*^02:01	25	Yes
	DQA1^*∗*^03:01			

DSA: donor-specific HLA antibody.

**Table 2:** HLA class II mismatch characteristics.

Mismatch ID	DSA	EpMS (ab, ot)	PIRCHE-II	HMS	EMS	AMS
1	No	4 (3, 1)	6	4.399	6.32	4
2	Yes	3 (1, 2)	8	4	4.749	4
3	Yes	8 (6, 2)	18	17.399	16	13
4	No	14 (4, 10)	9	12.499	14.229	16
5	Yes	9 (3, 6)	11	7.199	2.869	9
6	Yes	5 (2, 3)	9	5.499	10.969	9
7	Yes	16 (5, 11)	12	19.4	21.81	16
8	Yes	16 (9, 7)	12	25.499	19.1	19
9	Yes	15 (9, 6)	11	22.399	14.65	16
10	Yes	4 (3, 1)	12	19.899	20.79	10
11	No	13 (4, 9)	8	11.999	15.859	17
12	No	9 (1, 8)	7	14.3	15.43	11
13	Yes	12 (5, 7)	17	14.7	11.259	15
14	Yes	10 (2, 8)	13	3	1.03	7
15	Yes	12 (2, 10)	17	33.999	31.23	21
16	Yes	14 (4, 10)	9	12.499	14.229	16
17	No	6 (0, 6)	3	3.199	4.51	5
18	Yes	9 (3, 6)	11	7.199	2.869	9
19	No	6 (1, 5)	10	5.099	5.79	3
20	Yes	5 (2, 3)	9	5.499	10.969	9
21	Yes	11 (3, 8)	1	11.599	11.589	13
22	No	6 (1, 5)	3	10	10.03	8
23	Yes	8 (4, 4)	7	9	8.05	7
24	No	5 (2, 3)	2	0.9	0.529	3
25	Yes	4 (1, 3)	6	2	4.049	5

AMS: Amino acid mismatch score; DSA: donor-specific HLA antibody; EMS: electrostatic mismatch score; EpMS (ab, ot): eplet mismatch score (antibody verified and others); HMS: hydrophobicity mismatch score; PIRCHE-II: predicted indirectly recognizable HLA epitopes, HLA class II-presented.

**Table:** (a) HLA-DRB1 and DQA1/DQB1 mismatches

	No DSA (*n* = 8)	DSA (*n* = 17)	*P* value
EpMS	7.9 ± 3.8	9.5 ± 4.3	0.397
Antibody verified	2 ± 1.5	3.8 ± 2.4	0.0714
Others	5.9 ± 3	5.7 ± 3.1	0.8993
PIRCHE-II	6 ± 3	10.8 ± 4.3	**0.0094**
HMS	7.8 ± 5	13 ± 8.2	0.1438
EMS	9.1 ± 5.7	12.1 ± 8.1	0.3492
AMS	8.4 ± 5.7	11.6 ± 4.9	0.1544

**Table:** (b) HLA-DRB1 mismatches

	No DSA (*n* = 6)	DSA (*n* = 3)	*P* value
EpMS	8.7 ± 4.1	13.7 ± 2.5	0.0978
Antibody verified	2.2 ± 1.7	4 ± 1	0.1385
Others	6.5 ± 3.3	9.7 ± 1.5	0.1642
PIRCHE-II	6 ± 2.5	7.3 ± 5.7	0.6275
HMS	9.4 ± 4.6	14.5 ± 4.3	0.1516
EMS	11.1 ± 4.9	15.9 ± 5.3	0.2155
AMS	10.2 ± 5.5	15 ± 1.7	0.1919

**Table:** (c) HLA-DQA1/DQB1 mismatches

	No DSA (*n* = 2)	DSA (*n* = 14)	*P* value
EpMS	5.5 ± 0.7	8.6 ± 4.1	0.3248
Antibody verified	1.5 ± 0.7	3.7 ± 2.6	0.2707
Others	4 ± 1.4	4.9 ± 2.6	0.6679
PIRCHE-II	6 ± 5.7	11.5 ± 3.7	0.0836
HMS	3 ± 3	12.7 ± 9.8	0.1993
EMS	3.2 ± 3.7	11.3 ± 8.5	0.2105
AMS	3	10.9 ± 5.1	0.0524

*Note*. Values are expressed as mean ± SD; two-tailed *P* values are shown.

AMS: amino acid mismatch score; DSA: donor-specific HLA antibody; EMS: electrostatic mismatch score; EpMS: eplet mismatch score; HMS: hydrophobicity mismatch score; PIRCHE-II: predicted indirectly recognizable HLA epitopes, HLA class II-presented.

**Table 4:** Recipient's HLA-DR predicted presentation of self and nonself allo-HLA-derived peptide with highest affinity.

Recipient	Donor mismatches																													
	HLA-DRB1												HLA-DQB1												HLA-DQA1					
**TxID 13**	DRB1^*∗*^11:04												$\underline{DQB1^{*}03:01}$												$\underline{DQA1^{*}05:05}$					
	Self			Nonself									Self			**Nonself**									Self			**Nonself**		
	AA	IC_50_	% rank	AA	IC_50_	% rank							AA	IC_50_	% rank	AA	IC_50_	% rank							AA	IC_50_	% rank	AA	IC_50_	% rank

DRB1^*∗*^04:05	***158***	***1344***	***0.3***	47	6022	7							***158***	***2053***	***0.7***	11	3057	1.8							146	3369	2.5	***153***	***1032***	***0.15***
DRB1^*∗*^14:01	***158***	***2007***	***0.9***	47	3918	3.5							87	1426	0.5	***24***	***1203***	***0.4***							66	2605	1.5	***69***	***1813***	***0.7***

**TxID 18**	DRB1^*∗*^07:01												$\underline{DQB1^{*}02:02}$												$\underline{DQA1^{*}02:01}$					
	Self			Nonself									Self			**Nonself**									Self			**Nonself**		

DRB1^*∗*^11:01	***158***	***938***	***1.8***	6	1440	3							***87***	***600***	***1.1***	24	715	1.3							63	1465	3	***45***	***349***	***0.5***
DRB1^*∗*^16:01	158	3272	3.5	***6***	***2455***	***2.5***							122	1910	1.6	***47***	***1819***	***1.5***							148	1063	0.7	***45***	***1060***	***0.7***

**TxID 21**	$\underline{DRB1^{*}08:01}$												$\underline{DQB1^{*}03:01}$						$\underline{DQB1^{*}03:03}$						$\underline{DQA1^{*}06:01}$					
	Self			Nonself									Self			Nonself			Self			Nonself			Self			Nonself		

DRB1^*∗*^07:01	158	2199	2.5	***16***	***1167***	***0.8***							87	1908	1.9	***8***	***1286***	***1***	87	1908	1.9	***23***	***1532***	***1.3***	***148***	***2180***	***2.5***	54	2297	3

**TxID 22**	DRB1^*∗*^07:01						DRB1^*∗*^15:01						$\underline{DQB1^{*}02:02}$						$\underline{DQB1^{*}06:02}$						$\underline{DQA1^{*}02:01}$					
	Self			**Nonself**			Self			**Nonself**			Self			**Nonself**			Self			**Nonself**			Self			**Nonself**		

DRB1^*∗*^11:01	***158***	***938***	***1.8***	6	1440	3	***47***	***919***	***1.8***	88	1952	4.5	126	1848	4	***87***	***600***	***1.1***	126	1848	4	***87***	***109***	***0.08***	148	1774	4	***45***	***349***	***0.5***
DRB1^*∗*^14:07	***158***	***3491***	***1.2***	31	7990	9	***158***	***3491***	***1.2***	86	6554	5.5	16	6760	6	***83***	***2957***	***0.8***	16	6760	6	***87***	***1388***	***0.09***	140	5765	4.5	***69***	***2922***	***0.8***

**TxID 33**	$\underline{DRB1^{*}07:01}$						DRB1^*∗*^13:01						$\underline{DQB1^{*}02:02}$						DQB1^*∗*^06:04						$\underline{DQA1^{*}02:01}$					
	Self			Nonself			Self			Nonself			Self			Nonself			Self			Nonself			Self			Nonself		

DRB1^*∗*^11:01	***158***	***938***	***1.8***	6	1440	3	47	919	1.8	***24***	***526***	***0.9***	***87***	***600***	***1.1***	24	715	1.3	***87***	***166***	***0.17***	24	275	0.4	63	1465	3	***45***	***349***	***0.5***
DRB1^*∗*^16:01	158	3272	3.5	***6***	***2455***	***2.5***	47	2464	2.5	***24***	***2196***	***1.9***	122	1910	1.6	***47***	***1819***	***1.5***	***87***	***710***	***0.4***	24	1721	1.4	148	1063	0.7	***45***	***1060***	***0.7***

**TxID 35**	$\underline{DRB1^{*}07:01}$						DRB1^*∗*^15:01						$\underline{DQB1^{*}02:02}$						DQB1^*∗*^06:03						$\underline{DQA1^{*}02:01}$					
	Self			Nonself			Self			Nonself			Self			Nonself			Self			Nonself			Self			Nonself		

DRB1^*∗*^04:03	***157***	***1784***	***0.03***	37	9997	7	***158***	***1784***	***0.03***	67	13081	14	***158***	***4464***	***0.7***	47	7447	3.5	***158***	***4464***	***0.7***	87	7639	3.5	***153***	***5451***	***1.3***	69	6218	1.9
DRB1^*∗*^13:02	***159***	***3757***	***1.8***	30	5424	4	159	3757	1.8	***67***	***3244***	***1.3***	16	4547	2.5	***24***	***4083***	***2***	***16***	***4547***	***2.5***	87	6480	5	3	1055	0.15	***69***	***431***	***0.01***

*Note*. Bold and underlined mismatches led to the production of DSA. Bold amino acid position, IC50, and percentile rank are for the peptides with highest affinity for the recipient's HLA-DR antigens.

AA: amino acid position; % rank: percentile rank.

**Table 5:** Association between the nature (self or nonself) of the peptide predicted to bind with highest affinity to the recipient's HLA-II antigens and the presence of DSA.

Peptide length	HLA class II presentation	Allo-HLApep	DSA	No DSA	*P* value
9 mers	HLA-DR	Nonself	15	3	**0.0169**
		Self	2	5	
	HLA-DQ	Nonself	5	4	0.3942
		Self	12	4	
	Combined (HLA-DR and HLA-DQ)	Nonself	15	6	0.57
		Self	2	2	

12 mers	HLA-DR	Nonself	14	5	0.3442
		Self	3	3	
	HLA-DQ	Nonself	5	6	0.081
		Self	12	2	
	Combined (HLA-DR and HLA-DQ)	Nonself	14	8	0.527
		Self	3	0	

15 mers	HLA-DR	Nonself	14	6	1
		Self	3	2	
	HLA-DQ	Nonself	8	6	0.2337
		Self	9	2	
	Combined (HLA-DR and -DQ)	Nonself	15	8	1
		Self	2	0	

*Note*. Fisher's exact test two-tailed *P* values are shown.
